# Pupillometric parameters of alertness during unpredictable but not predictable smooth pursuit neck torsion test are altered in patients with neck pain disorders: a cross-sectional study

**DOI:** 10.1007/s00221-023-06648-z

**Published:** 2023-07-15

**Authors:** Ziva Majcen Rosker, Grega Mocnik, Eythor Kristjansson, Miha Vodicar, Jernej Rosker

**Affiliations:** 1 Faculty of Sport, University of Ljubljana, 1000 Ljubljana, Slovenia; 2 Laboratory for Digital Signal Faculty of Electrical Engineering and Computer Science, University of Maribor, 2000 Maribor, Slovenia; 3 Landspitali University Hospital, 101 Reykjavik, Iceland; 4 Chair of Orthopaedics, Medical Faculty, University of Ljubljana, 1000 Ljubljana, Slovenia; 5 Department of Orthopaedic Surgery, University Medical Centre Ljubljana, 1000 Ljubljana, Slovenia; 6 Faculty of Health Sciences, University of Primorska, 6310 Izola, Slovenia

**Keywords:** Oculomotor functions, Smooth pursuit eye movements, Cervical disorders, Cognitive disfunction, Attention, Pupillometry

## Abstract

Despite commonly investigated predictable smooth-pursuit neck-torsion tasks (SPNT) in neck pain patients, unpredictable conditions have been seldom investigated but are indicative of preserved oculomotor functions during neck torsion. Although not previously studied, some speculations about compensatory cognitive mechanisms such as increased phasic alertness during unpredictable tasks were suggested. The aim of this study was to investigate eye movement accuracy and pupillometric responses during predictable and unpredictable SPNT test in neck pain patients and asymptomatic controls. Eye movements (gain and SPNT-difference) and pupillometry indicative of tonic (average and relative pupil diameter) and phasic (index of cognitive activity-ICA) alertness were measured in 28 idiopathic neck pain patients and 30 asymptomatic individuals using infrared video-oculography during predictable and unpredictable SPNT test. Gain in unpredictable SPNT test was lower as compared to predictable tasks and presented with similar levels in neutral and neck torsion positions, but not in the predictable SPNT test. ICA was lower during neutral position in all tasks in patients as compared to control group but increased during neck torsion positions in unpredictable tasks. Relative pupil diameters presented with no differences between the groups or neck positions, but the opposite was observed for average pupil diameter. Higher ICA indicates an increase in phasic alertness in neck pain patients despite no alterations in oculomotor control during SPNT test. This is the first study to indicate cognitive deficits in oculomotor task in neck pain patients. The latter could negatively affect other tasks where additional cognitive resources must be involved.

## Introduction

Patients with neck pain disorders commonly present with disturbances in the oculomotor system (Tjell and Rosenhall 1998; Treleaven et al. 2005, 2011; Majcen Rosker et al. 2022b) of which the goal is to maintain visual information carriers’ retinal projection on or near the fovea during visual field observation (Fukushima et al. 2013; Brostek et al. 2017). Such disturbances in neck pain patients are reflected in decreased ability to smoothly pursuit a horizontally moving target with their eyes, especially when the neck is in torsioned position (SPNT) (Tjell and Rosenhall 1998; Tjell et al. 2002).

The proposed mechanism for deficiencies in eye movement control during neck torsion position is error in proprioceptive drive leading to disturbances in cervico-colic and cervico-ocular reflexes (Tjell and Rosenhall 1998; Kristjansson and Treleaven 2009). Such adaptations could result in decreased ability to track a horizontally moving target with their eyes in neck torsion positions, expressed as increased difference between precision of smooth pursuit eye movements (gain) during neutral and neck torsion positions (SPNTdiff) (Tjell et al. 2002).

In addition to above described relevance of cervical sensory information in oculomotor control, target projection slippage on the retina or its distance from the fovea supplements extraocular muscles activity and consequently ocular movements (Brostek et al. 2017). These information are used by the central eye movement controlling mechanisms for anticipation of target movement characteristics such as spatial and temporal characteristics of target movement trajectory (Brostek et al. 2017). However, online eye movement corrections must supplement anticipatory eye movements, especially during unpredictable target movements. Such corrections are time consuming and can lead to less accurate smooth pursuit eye movements (Haarmeier and Thier 2006).

As suggested by Majcen Rosker et al. (2022b) different characteristics of target movement, such as increased velocity or amplitude, could influence difficulty of smooth pursuit eye movement tasks in neck pain patients. For example, smoothly pursuing a target at higher velocities increases activity of saccadic system causing decreased accuracy of eye movement (Land 2006), while amplitude of eye movements affects neck muscle activity (Bexander et al. 2005) possibly adding towards commonly observed sensory mismatch (Liu et al. 2021). Therefore, as described above it could be expected, that unpredictably changing target movement amplitude or velocity could present an increased challenge to oculomotor control as compared to predictable target movements.

When the difficulty of following a moving target increases, cognitive resources such as working memory and attention are deemed more involved (Haarmeier and Thier 2006). According to research, neck pain patients present with alterations in eye movements when observing predictable horizontally moving target (Treleaven et al. 2005; Majcen Rosker et al. 2022a, b), but to our knowledge only one study analysed eye movement control during unpredictable target movements at neutral and neck torsion positions (Janssen et al. 2015). Their results showed preserved eye movements during the neck torsion manoeuvre while tracking an unpredictable but not predictable target. The authors speculated that smooth pursuit eye movement performance increase during neck torsion manoeuvre in unpredictable task might have resulted from altered cognitive involvement, especially increased level and changed type of alertness. Cognitive disfunctions, that also involve alertness, are commonly described by neck pain patients (Gimse et al. 1996, 1997b; Bosma and Kessels 2002; Borenstein et al. 2010). Amongst others, altered ability to concentrate to read or focus and difficulty judging distance are commonly reported (Treleaven and Takasaki 2014). Moreover, relationship between above mentioned symptoms and SPNT test has been observed (Majcen Rosker et al. 2022c). It is currently unknown whether cognitive deficits such as altered alertness are present during predictable and unpredictable oculomotor tasks and to what extent is alertness altered in neck pain patients. It would be important to understand whether they can mobilize supplementary cognitive resources when performing predictable and unpredictable SPNT tasks. Such adaptations could importantly decrease capacity of otherwise limited cognitive resources (Land 2006).

Alertness during visual tasks is commonly assessed using pupillometry, which has been shown to be an objective and reliable method (Vogels et al. 2018; Zele and Gamlin 2020). Pupillary dilatations are suggested to result from increased activity of locus coeruleus (Marshall 2000, 2007; Czerniak et al. 2021), which is related to level of alertness (Moazen et al. 2020) and can be altered in presence of pain (Moazen et al. 2020). In general, alertness measured via pupillary responses can be divided into the slow adapting pupil dilatations representing tonic alertness (attending to various objects simultaneously) and high frequency pupillary responses representing phasic alertness (attending to a specific object) (O’Bryan and Scolari 2021). The aim of this study was to analyse the effect of neck torsion on the ability to smoothly follow predictable and unpredictable moving targets in neck pain patients and asymptomatic individuals. Moreover, accompanied changes in tonic and phasic alertness were studied to better understand cognitive compensatory mechanisms during eye movements tasks at neck torsion manoeuvre in neck pain patients.

## Materials and methods

### Participants

A group of chronic neck pain patients and asymptomatic controls participated in this study. Asymptomatic controls were enrolled among university staff members and their acquaintances. Patients with neck pain for longer than 6 months were recruited at orthopaedic outpatient clinics. All participants were required to present with minimum of 50° of cervical rotation to each side and had to be in an age range 18–55 years. Patients were required to mark pain intensity on 10-cm horizontal line of visual analogue scale (Boonstra et al. 2014) presenting with minimum of 4 to be considered for the study. All participants had to be free from previous traumatic injury to the neck or head, shoulders or upper extremities pain, any neurological or vestibular disorders, and were required to take no medication or alcohol for 30 h before the study. In addition, all patients presented with results of magnetic resonance imaging. The study was approved by the national medical ethics committee (No. 0120-47/2020/6) and was performed in accordance with the declaration of Helsinki.

### Equipment

A 100-Hz infrared video-oculography (Pro Glasses 2, Tobii, Danderyd, Sweden) was used to measure eye movements during SPNT test and left eye pupillary diameter (Piñero et al. 2020). Participants were instructed to track a horizontally moving target of a red dot (size 0.5° of visual angle) which was projected with a 100-Hz refresh rate (Optoma ML1050ST LED Projector, Fremont, USA) 150 cm away at an eye level (Deravet et al. 2018). Participants were sitting on a custom-made rotatable chair with upper body fixed to the back support. All measurements were conducted by the same examiner in a room with constant illumination.

### Experiment

Testing protocol consisted of four horizontal SPNT tests of which characteristics were based on previous studies (Majcen Rosker et al. 2021, 2022d); (i) tracking a predictable cyclic sinusoidal target movement with 40° target movement amplitude and 30°s^−1^ target movement velocity (predictable SPNT test), (ii) tracking a sinusoidal target movement with changing target movement amplitude ranging from 30° to 50° amplitude at constant velocity of 30°s^−1^ SPNT test (unpredictably changing amplitude), (iii) tracking a sinusoidal target movement with changing target movement velocity ranging from 20 to 40°s^−1^ at a constant target movement amplitude of 30° (unpredictably changing velocity) and (iv) a sinusoidal target movement with changing of amplitude (from 30° to 50° amplitude) and velocity (from 20 to 40°s^−1^) (unpredictably changing amplitude and velocity—unpredictable task).

All four tasks were performed at three neck positions: (i) neutral position with the trunk and head facing forward, (ii) torsion of the neck for 45° to the left (rotation of the trunk underneath the stationary head to the right) and (iii) torsion of the neck for 45° to the right (rotation of the trunk underneath the stationary head to the left). The order of neck torsions was pseudo-randomized across subjects.

Subjects were required to track 10 cycles of sinusoidal target movements followed by 60 s rest with all tasks performed in random order.

### Data analysis

Eye movement data were filtered for blinks, saccades and fixations using Tobii Pro Lab software (Tobii Pro lab 1.145, Tobii, Danderyd, Sweden). Square waves (saccades directed counter to each other and having an interval of relative standstill) were removed from eye movement data using custom-written software in MATLAB (R2017b, MathWorks, Natick, MA, USA). Eye movement data were fitted with a corresponding reference sinusoid. Each fitted sinusoid consisted of 10 cycles with corresponding amplitude (converted from angular degrees to pixels) and frequency matching the profile for each individual condition. Horizontal eye movements were analysed using gain, calculated as the ratio between fitted eye velocity amplitude and visual target velocity amplitude as described by Tjell et al. (2002). Gain torsion R represents the average gain during the right neck torsion and gain torsion L represents the average gain during left neck torsion from the 6th to 9th cycle (Majcen Rosker et al. 2022a). Additionally, SPNTdiff was calculated as presented in Eq. 1 to present differences between neutral and neck torsion positions. The calculation was adapted and is similar as described by Tjell et al. (2002).

1 $${\text{SPNT}}_{{{\text{diff}}}} = {\text{Gain neutral}} - \left( {{\text{Gain torsion L}} + {\text{Gain torsion R}}} \right)/{2}$$

Equation (1): gain neutral represents the average gain in the neutral position from the 6th to 9th cycle, gain torsion L represents the average gain during the left neck torsion position from the 6th to 9th cycle and gain torsion R represents the average gain during the right neck torsion position from the 6th to 9th cycle.

Pupil size data were analysed using two approaches. The index of cognitive activity (ICA) was derived from the pupil size data using a procedure described in Marshall (2000). This procedure is performed on pre-prepared data, where short blinks are interpolated to obtain a continuous pupil size data set. Furthermore, Wavelet analysis was used to decompose the pupil signal into high-frequency components which are representative of changes in cognitive activity. Rapid pupil dilatations exceeding a threshold are identified and used to calculate the ICA. The procedure was patented in 2000 (US Patent Number 6.090.051) and the values can be obtained via the Cognitive Workload Module (Cognitive Workload Module 3, EyeWorks, San Diego, USA). The software provides a number of pupil dilatations per second, normalizes and transforms them (Marshall 2000, 2007). The ICA was averaged over 6th–9th cycle of each unpredictable and predictable task. In addition, average pupil size was calculated during 6th–9th cycle of the unpredictable and predictable tasks. The average pupil diameter at each unpredictable task was further expressed as a ration between the average pupil diameter during unpredictable and predictable SPNT tests (relative pupil diameter) (Zénon 2019). Average pupil diameter and relative pupil diameter were used for further analysis.

### Statistical analysis

Statistical analysis was performed in SPSS (SPSS 23.0 software, SPSS Inc., Chicago, USA). Shapiro–Wilk test, skewness and kurtosis were calculated to analyse data distribution for each parameter. Median and interquartile range were calculated for both groups in each test and neck position. Due to non-normality of data distribution in some parameters, Friedman’s test was used to analyse differences between the neck positions in each SPNT tasks for each group separately and for differences between tests for each position and group separately. Post-hoc sign rank test was used for pairwise comparisons. Differences between groups were analysed using Sign test for each neck position and each SPNT test separately. Cohen d was calculated for each post-hoc test. Statistical significance was set at *p* < 0.05.

## Results

### Participants

Twenty-eight patients and thirty controls were recruited for the study. Twenty-one women and seven men were included in the patient group and nineteen women and eleven men in the control group. The mean age of the patient’s group was 42.1 ± 4.6 years (age range 27–51 years) and the mean age of the control group 39.3 ± 5.7 years (age range 23–50 years). The control group was statistically significantly older as compared to the patient group (*p* = 0.046; *d* = 0.203). In the neck pain group cervical spine magnetic imaging assessment presented disc protrusions or herniations at levels from C4 to Th1 in 23 patients, seven patients presented with facet joint osteoarthritis at the levels from C5 to Th1, six patients with low-grade spondylolisthesis and seven patients with cervical spinal stenosis. Nineteen patients had a combination of at least two types of structural deformity, but only one was present in nine patients. Average pain duration in the patient’s group was 11.3 ± 6.9 months and average VAS score was 4.9 ± 1.8. Control group presented with no pain.

### Neck position and group differences

Table 1 presents the results of the Friedman’s test where the differences in gain, ICA, average pupil diameter and relative pupil diameter between the three neck positions were analysed at each SPNT tests for both groups separately. Statistically significant differences were present only for Gain in the predictable SPNT test and ICA in all three unpredictable tasks for patients with neck pain.

**Table 1:** Results of Friedman’s test

Parameter	SPNT task	Neck pain patients	Healthy controls
		*χ*^2^	*χ*^2^
Gain	Predictable	4.392*	1.849
	Unpredictable	3.457	2.378
	Unpredictable amplitude	2.918	4.014
	Unpredictable velocity	1.962	1.381
Index of cognitive activity	Predictable	2.286	3.271
	Unpredictable	5.286*	1.254
	Unpredictable amplitude	5.143*	1.857
	Unpredictable velocity	6.001*	4.308
Average pupil diameter	Predictable	0.182	2.462
	Unpredictable	0.149	2.462
	Unpredictable amplitude	1.077	4.429
	Unpredictable velocity	0.247	4.154
Relative pupil diameter	Unpredictable	0.220	3.659
	Unpredictable amplitude	0.176	2.974
	Unpredictable velocity	0.414	3.185

SPNT test smooth pursuit neck torsion test, *χ*^2^ Chi-square statistic

*Statistical difference > 0.05

### Pair-vice comparisons for gain

**Fig. 1:**
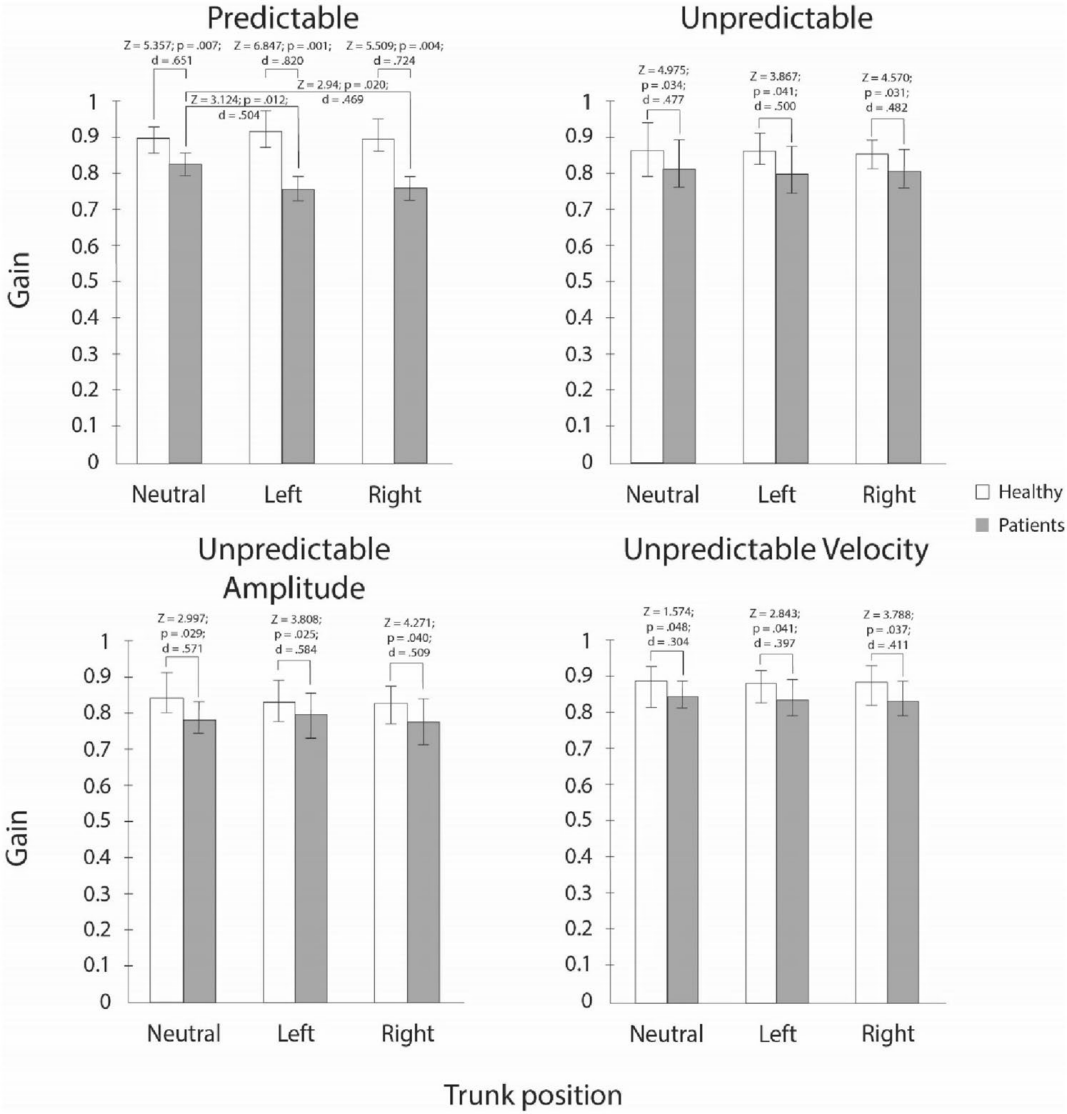
Gain

Medians, interquartile ranges, and results of the sign post-hoc tests for differences in gain between two group and neck torsion position are presented in Fig. 1. The two groups differed statistically significant in all SPNT tests and neck torsion positions observed. Statistically significant differences between neutral and both neck torsion positions were observed only in patient group in predictable SPNT test.

### Pair-vice comparisons for SPNTdiff

**Fig. 2:**
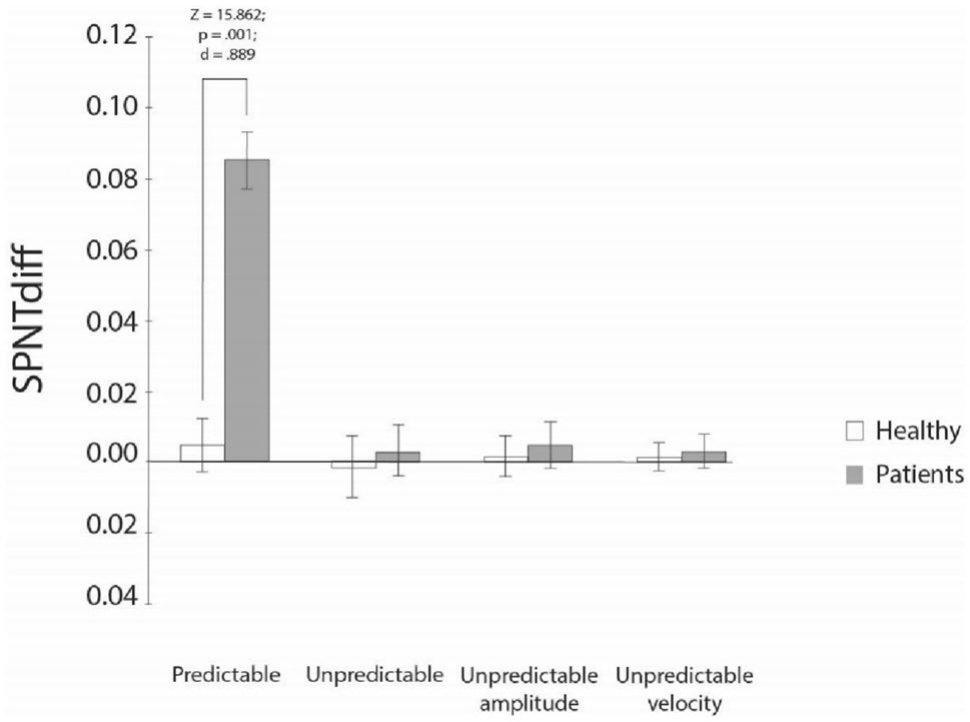
Smooth pursuit neck torsion difference. SPNT_diff_ smooth pursuit neck torsion difference, *z* *z* statistics, *p* statistical difference, *d* Cohens *d*

Medians, interquartile ranges, and results of the sign post-hoc tests for differences in the SPNTdiff for group and neck torsion position are presented in Fig. 2. Statistically significant differences were observed for SPNTdiff in the predictable but not for three unpredictable SPNT tests.

### Pair-vice comparisons for ICA

**Fig. 3:**
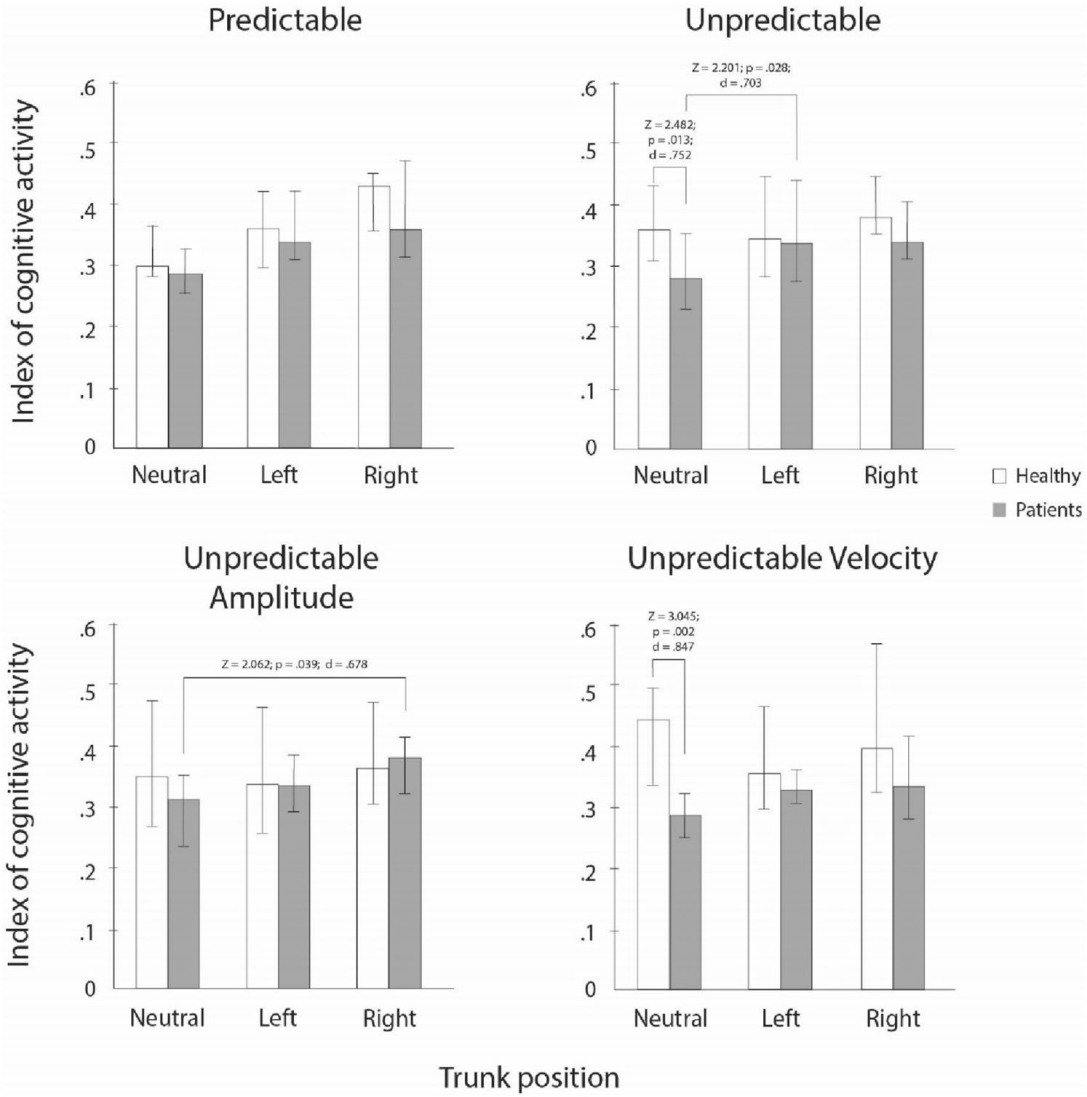
Index of cognitive activity. *Z* *z* statistic, *p* statistical significance, *d* Cohens *d*

Medians, interquartile ranges, and results of the sign post-hoc tests for differences in the ICA for group and neck torsion position are presented in Fig. 3. Statistically significant differences between groups were observed for the unpredictable SPNT test and unpredictable SPNT test with varying velocity in the neutral neck position. Differences between neutral and some neck torsion positions were observed in unpredictable SPNT test and unpredictable SPNT test with varying amplitude in patient group. In control group, no statistically significant differences were observed.

### Pair-vice comparisons for average and relative pupil diameter

**Fig. 4:**
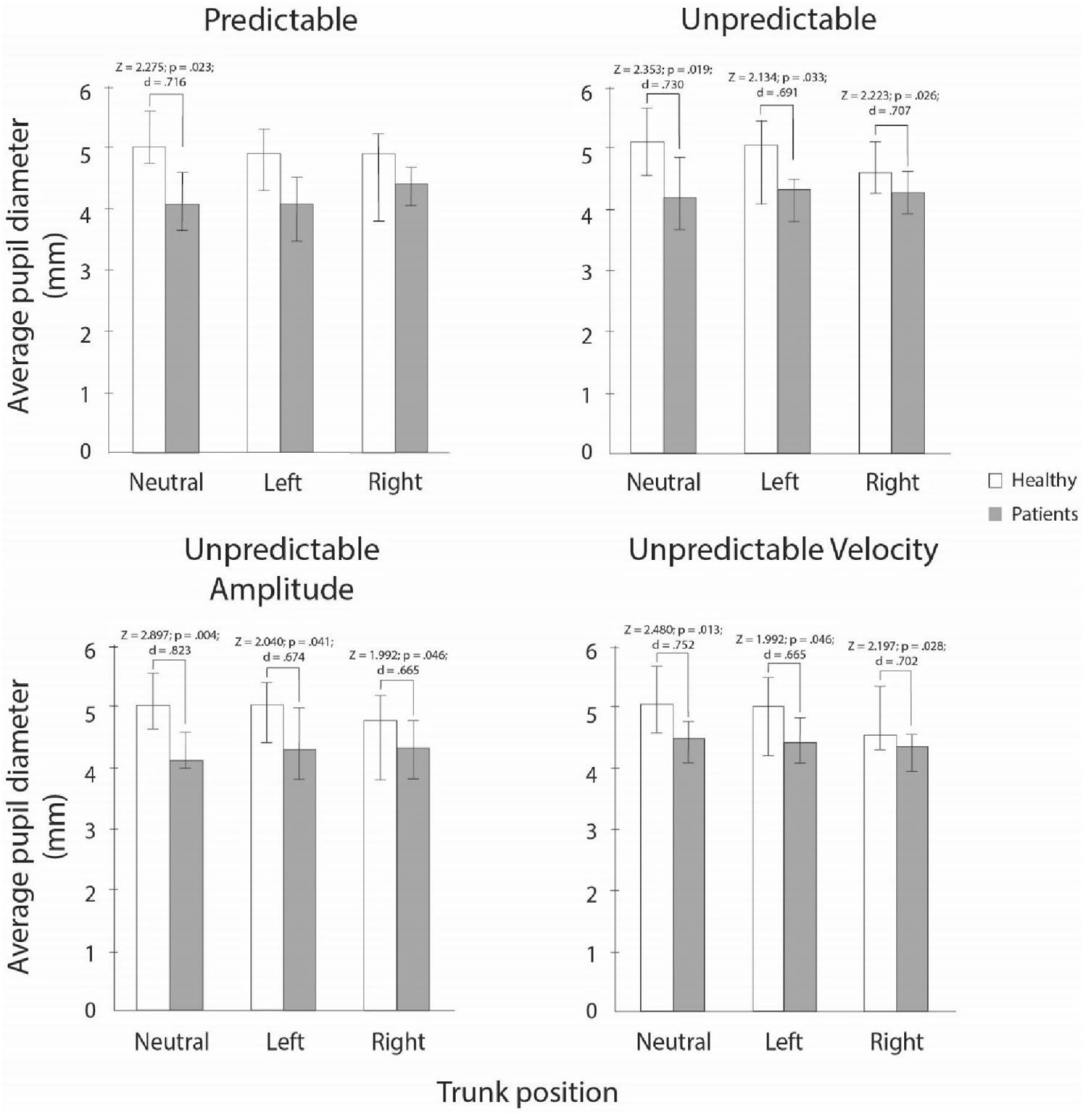
Average pupil diameters. *Z* *z* statistic, *p* statistical significance, *d* Cohens *d*

Medians, interquartile ranges, and results of the sign post-hoc tests for differences in the average pupil diameter for both groups and neck torsion position are presented in Fig. 4. Statistically significant differences between both groups were observed for the predictable SPNT test in neutral neck position and for all neck positions in all three unpredictable SPNT test. No statistically significant differences between neck positions were observed for both groups.

**Fig. 5:**
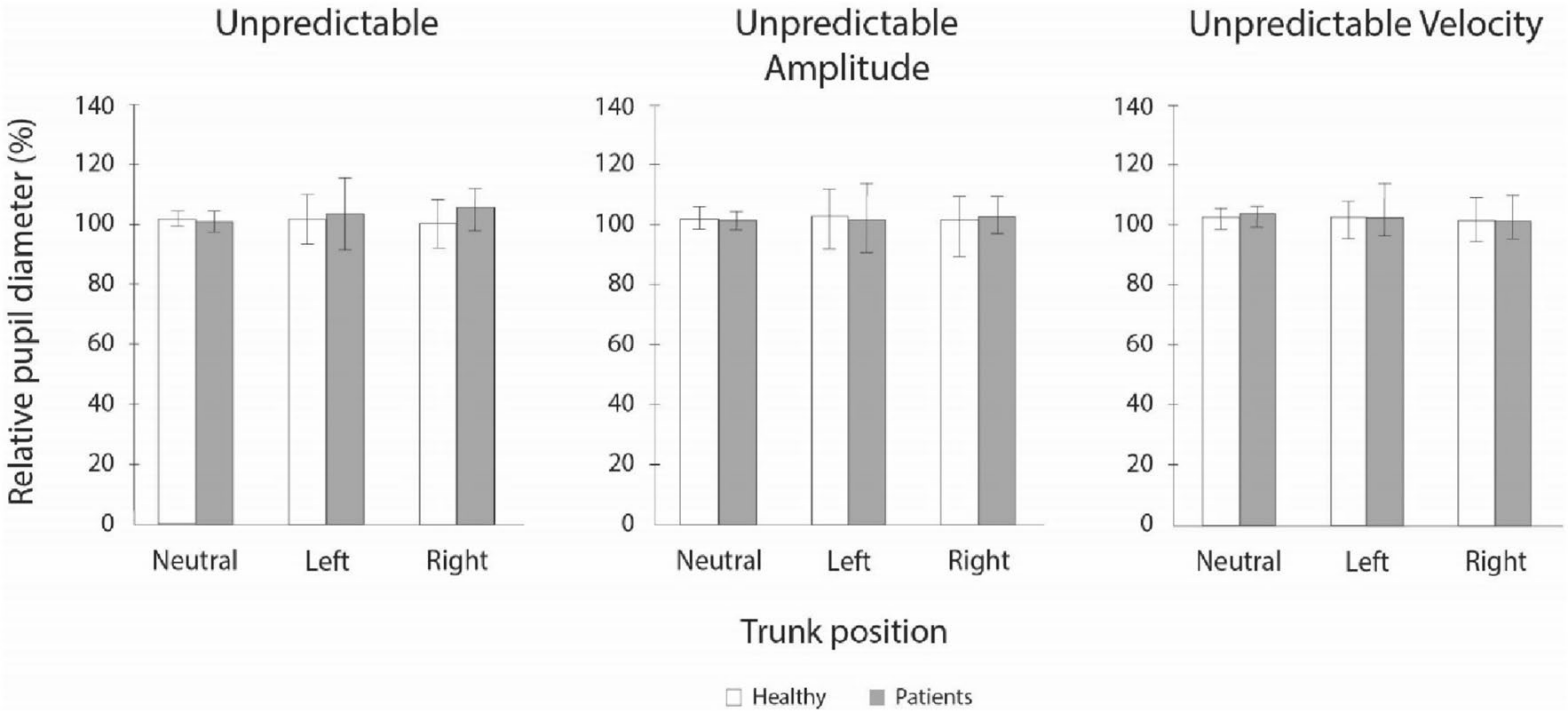
Relative pupil diameter

Medians, interquartile ranges, and results of the sign post-hoc tests for differences in relative pupil diameter for both groups and neck torsion positions are presented in Fig. 5. No statically significant differences were observed between the groups as well as between three neck torsion positions.

## Discussion

The aim of this study was to compare performance of SPNT test using one predictable and three unpredictable target movement profiles (varying target movement amplitude, velocity or both simultaneously) in neck pain patients and asymptomatic individuals. Additionally, possible changes in tonic and phasic alertness were assessed during all SPNT tests for both studied groups. Neck pain patients presented with decreased precision (decreased gain) to follow a moving target in all SPNT tests as compared to asymptomatic individuals. Moreover, during predictable target movements patients presented with decreased gain (higher SPNTdiff) in neck torsion positions as compared to the neutral position, which was not observed in unpredictable target movement tasks (lower SPNTdiff). Higher ICA presented with an increase in alertness under neck torsion manoeuvre as compared to the neutral position in neck pain patients. This was evident for unpredictable and unpredictable SPNT test with varying amplitude (left and right torsion, respectively) but not predictable SPNT test. Although similar trend was observed for predictable SPNT test, it could be speculated that this was due to its lesser challenge to the cognitive system. On the contrary, asymptomatic individuals presented with similar alertness in the neutral and neck torsion positions. Comparisons between the two groups presented with statistically significant differences in ICA but only during tasks in the neutral position. Tonic alertness presented with statistically significant differences between groups for all observed tasks and neck positions when observing the average pupil diameter, but not for the relative pupil diameter. Moreover, no differences between neck positions were observed for both parameters of alertness for either of the groups.

Although previous studies investigating predictable eye movement tasks indicate that amplitude and velocity might play an important role in the accuracy of eye movements (Bexander et al. 2005; Land 2006; Majcen Rosker et al. 2022b), this has not been the case when observing unpredictable SPNT tests. To our knowledge study performed by Janssen et al. (2015) was the only study investigating SPNT test performance during unpredictably changing velocity of target movements. Our study aimed to determine whether unpredictably changing amplitude, velocity or both would influence the results of SPNT test differently. Results from our study add to current knowledge that unpredictable changes in target movement amplitude, velocity or both present with no differences in gain in neck pain patients indicating that target movement amplitude or velocity do not play as significant role in unpredictable SPNT tests.

Gain in predictable SPNT tests observed in our study was in line with the results reported by other studies (Tjell and Rosenhall 1998; Treleaven et al. 2005; Majcen Rosker et al. 2022a), where a decrease in eye movement accuracy was observed in neck torsion position as compared to neutral position, leading to increase in SPNTdiff. Interestingly gain in unpredictable tasks reported in our study remained unchanged in neck torsion positions. Our results are in line with previous findings presented by Janssen et al. (2015) where neck torsion positions showed no alterations in gain as compared to neutral position. In general, decreased gain under neck torsion position in predictable SPNT tests is suggested to result from sensory mismatch caused by altered sensory drive from the impaired cervical spine, projecting to superior colliculus and influencing vestibular and visual systems (Peterson 2004; Cheever et al. 2016). As a consequence of sensory mismatch, cervico-colic and cervico-ocular reflexes are altered, causing decreased accuracy of eye movement control during neck torsion positions (Tjell et al. 2002; Majcen Rosker et al. 2022b). The above-described mechanism of eye movement control could be less prevailing during unpredictable SPNT tests due to involvement of higher order mechanisms governing eye movements (Fukushima et al. 2013; Brostek et al. 2017). Retinal slippage or distance of the retinal target projection from the fovea are supposed to be important sources of information controlling eye movements (Haarmeier and Thier 2006; Tavassoli and Ringach 2010). During more demanding SPNT tests (unpredictable target movements) such information on previous target movement influences anticipatory eye movements enabling compensations for delays in sensory feedback loops. These mechanisms are supposed to be governed by higher order processing in the frontal eye fields which demands involvement of cognitive resources such as visual working memory and alertness (attention) (Brostek et al. 2017). Higher order systems could efficiently compensate for the presence of sensory mismatch caused by cervical disfunction. This could explain the results from our study as well as results presented by Janssen et al. (2015), where gain during neck torsion remained at the comparable level as during neutral position.

Neck pain patients commonly present with cognitive, more specifically alertness deficits (Thompson et al. 2010; Takasaki et al. 2013). The increased allocation of the cognitive resources to the SPNT tests under unpredictable conditions was suggested to be the cause of improved gain during neck torsion positions in neck pain patients (Janssen et al. 2015). This suggestion was partially confirmed by our study, where ICA, which is supposed to be related to object-target specific attention allocation (tonic alertness) (O’Bryan and Scolari 2021), was increased under neck torsion position. In addition, the ICA was in general decreased in the neutral neck position as compared to healthy controls, which confirms the presence of phasic alertness deficit in patients with neck pain disorders as compared to asymptomatic individuals. The main difference was that in the predictable SPNT test there were no statistically significant differences in ICA between the neutral and neck torsion positions. This suggests that predictable SPNT task was not cognitively challenging enough which could expose possible effects of proprioceptive deficits on eye movement control. Under neck torsion conditions difficulty of tasks increased, demanding increased alertness to focus on the moving target and perceive target movement changes, which could have compensated oculomotor deficits on the expense of increased involvement of cognitive resources. This observation is important to understand the challenge of everyday tasks in neck pain patients. During more demanding visual tasks, neck pain patients are likely to be better able to compensate for oculomotor deficits, however, their cognitive capacity is consequently decreased, making less cognitive resources available for other tasks. Such alterations in cognitive resources could influence other skills where vision is important (e.g. driving a car, walking in a crowded environment, performing reading tasks where additional cognitive resources are demanded) (Gimse et al. 1996, 1997a, b). This could lead to earlier fatigue development and decreased general ability to perform more cognitive demanding work.

Somewhat expected, tonic alertness expressed as a relative pupil diameter, did not show any specific differences between the two groups as well as between neck positions for either of the groups. Tonic alertness is thought to be involved in attending to multiple sources of information simultaneously. In our study during SPNT task only one stimulus (target) was used, with all additional sources of information omitted from the visual field. On the contrary, tonic alertness described by an average pupil diameter was statistically significantly lower in neck pain patients as compared to asymptomatic individuals in all studied tasks and neck positions. This suggests possible impairments of tonic alertness in neck pain patients as compared to asymptomatic individuals.

Although our results indicate alertness alterations in neck pain patients, more studies are needed to confirm our observations. An important limitation of our study was that it is unclear to what extent the pupil diameter could have been affected by posturally modulated activity of locus coeruleus. Changes in neck position have been shown to influence the activity of locus coeruleus in animals (Pompeiano et al. 1991). The latter is suggested to be related to adaptations in sensory-motor control at the level of brainstem. It is, however, unknown whether activity of locus coeruleus and consequently pupillary responses can be modulated by changes in neck position in humans, and whether this relation is affected by cervical deficits. Therefore, it is unknown whether ICA, average and relative pupil diameter can be interpreted solely as measures of cognitive functions or alertness when performing tasks in neck torsion position.

An additional limitation of this study was that the intake of stimulants such as coffee was not controlled. This could have influenced alertness in some participants and consequently added towards increased variability between the two groups. However, it is not possible to hypothesize whether use of stimulants would affect differences between neck torsion and neutral position and three different SPNT tasks in either of groups.

## Conclusion

The results of our study confirm previous results showing that neck pain patients are able to compensate for oculomotor deficits in neck torsion position during unpredictable smooth pursuit eye movement tasks using higher order cognitive and oculomotor control processes. Both tonic and phasic alertness have been shown to be altered during smooth pursuit eye movement tasks, however, phasic alertness was the primary compensatory mechanisms which enables neck pain patients to preserve precision of oculomotor control during neck torsion positions. These results help to better understand the wider effect of neck pain on other aspects of patient’s daily activities and consequently help to better understand the interconnection between different signs and symptoms.

## Data Availability

The datasets generated during and/or analysed during the current study are available from the corresponding author on reasonable request.
